# Supplementary material for: Therapeutic high affinity T cell receptor targeting a KRASG12D cancer neoantigen
Source: Nat Commun. 2022 Sep 10;13:5333. doi: 10.1038/s41467-022-32811-1 (PMC9464187; doi:10.1038/s41467-022-32811-1)
Supplement: Supplementary file 1 — Supplementary Information [file 41467_2022_32811_MOESM1_ESM.pdf]

Therapeutic high affinity T cell receptor targeting a $KRAS^{G12D}$ cancer neoantigen

Supplementary Information

SUPPLEMENTARY FIGURES

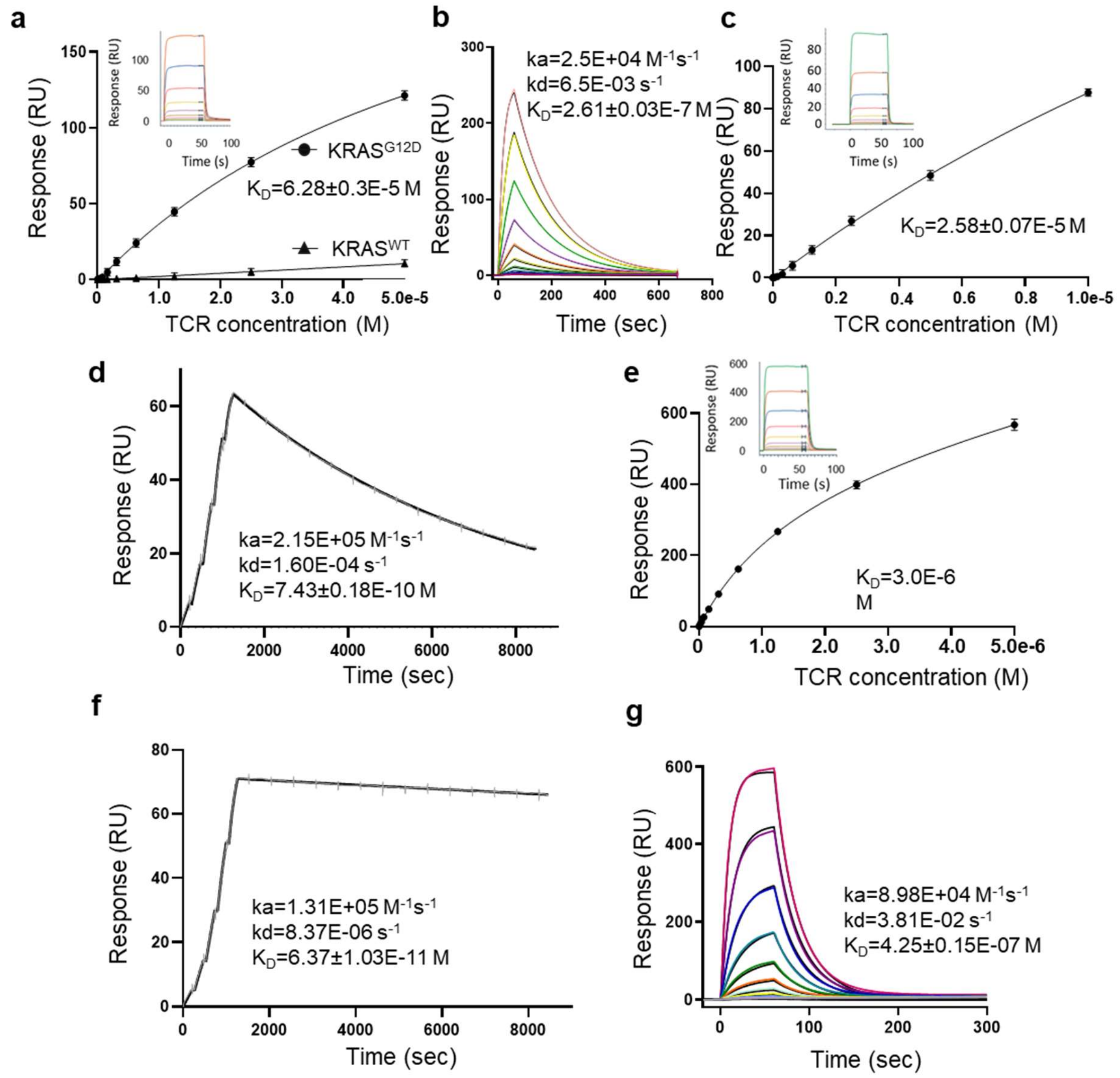

Fig S1. Affinity (K_D) measurement of JDI, JDIa41b1 and IMC-KRAS^{G12D} TCR binding to HLA-A*11-KRAS^{G12D} and HLA-A*11-KRAS^{WT}. **a**. JDI TCR steady state binding analysis using ten 2-fold dilutions starting at highest concentration of 50 µM. **b**. Multicycle kinetic analysis of JDIa9bwt TCR binding to HLA-A*11-KRAS^{G12D} measured using ten 2-fold dilutions starting at top concentration of 2µM. **c**. Steady state binding analysis of JDIa9bwt binding to HLA-A*11-KRAS^{WT} measured using ten 2-fold dilutions starting at top TCR concentration of 10 µM. **d**. Single cycle kinetic analysis of JDIa41b1 TCR binding to HLA-A*11-KRAS^{G12D} using 5 injections at 2.5 nM, 5.0 nM, 10 nM, 20 nM and 40 nM TCR concentration. **e**. Steady state binding analysis of JDIa41b1 TCR binding to HLA-A*11-KRAS^{WT} measured using ten 2-fold dilutions starting at highest TCR concentration of 5 µM.

f. Single cycle kinetic analysis of IMC-KRAS^{G12D} binding to HLA-A*11-KRAS^{G12D} using 5 injections at 2.5 nM, 5.0 nM, 10 nM, 20 nM and 40 nM TCR concentration. **g**. Multicycle kinetic analysis of IMC-KRAS^{G12D} TCR binding to HLA-A*11-KRAS^{WT} measured using ten 2-fold dilutions starting at highest TCR concentration of 1 μM. SPR sensorgrams are shown in inset for a, c and e. Experiments were replicated (n=2 for a, d and n=3 for b, c, e, f, g) and the K_D values are mean ± SD. Source data are provided as a Source Data file.

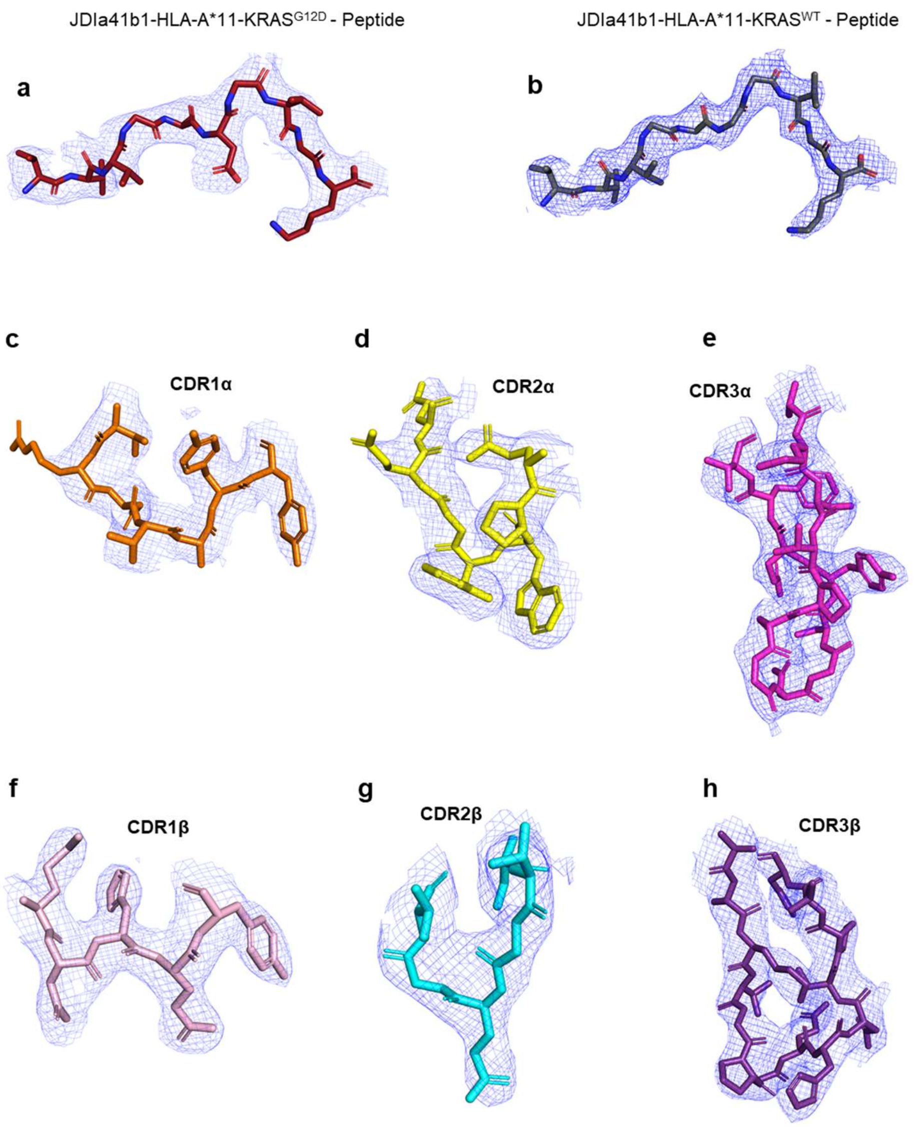

Fig S2. Composite omit maps (2Fo-Fc) of KRAS peptides and JDIa41b1 TCR CDR regions. a. Composite omit map of KRAS^{G12D} peptide in the JDIa41b1-HLA-A*11-KRAS^{G12D} (PDB 7OW6) complex**. b.** Composite omit map of KRAS^{WT} peptide in the JDIa41b1-HLA-A*11-KRAS^{WT} complex (PDB 7OW5). **c-h.** Composite omit maps of JDIa41b1 TCR alpha and beta chain CDR regions in the JDIa41b1-HLA-A*11-KRAS^{G12D} complex. The maps are contoured at contoured at 1σ with carve radius of 1.8 Å. The peptides and CDR regions are coloured as in figure 2.

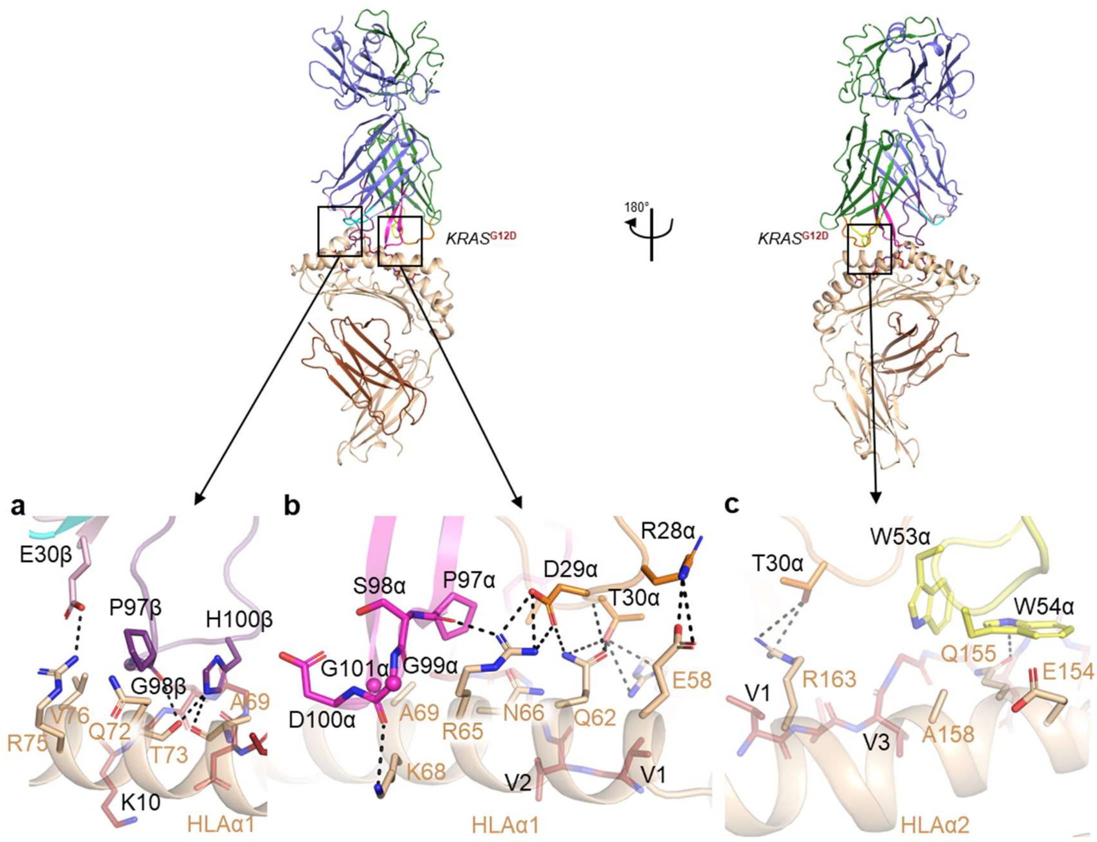

Fig S3. TCR and HLA interaction network in the JDIa41b1-HLA-A*11-KRAS^{G12D} (PDB 7OW6) complex. a. Interactions between the JDIa41b1 TCRβ CDRs and HLA. **b-c.** Interactions between the JDIa41b1 TCRα and HLA. The TCR and HLA residues that are up to 4 Å of each other are shown in sticks. The dotted lines indicate polar contacts. The KRAS^{G12D} peptide residues are shown as maroon sticks.

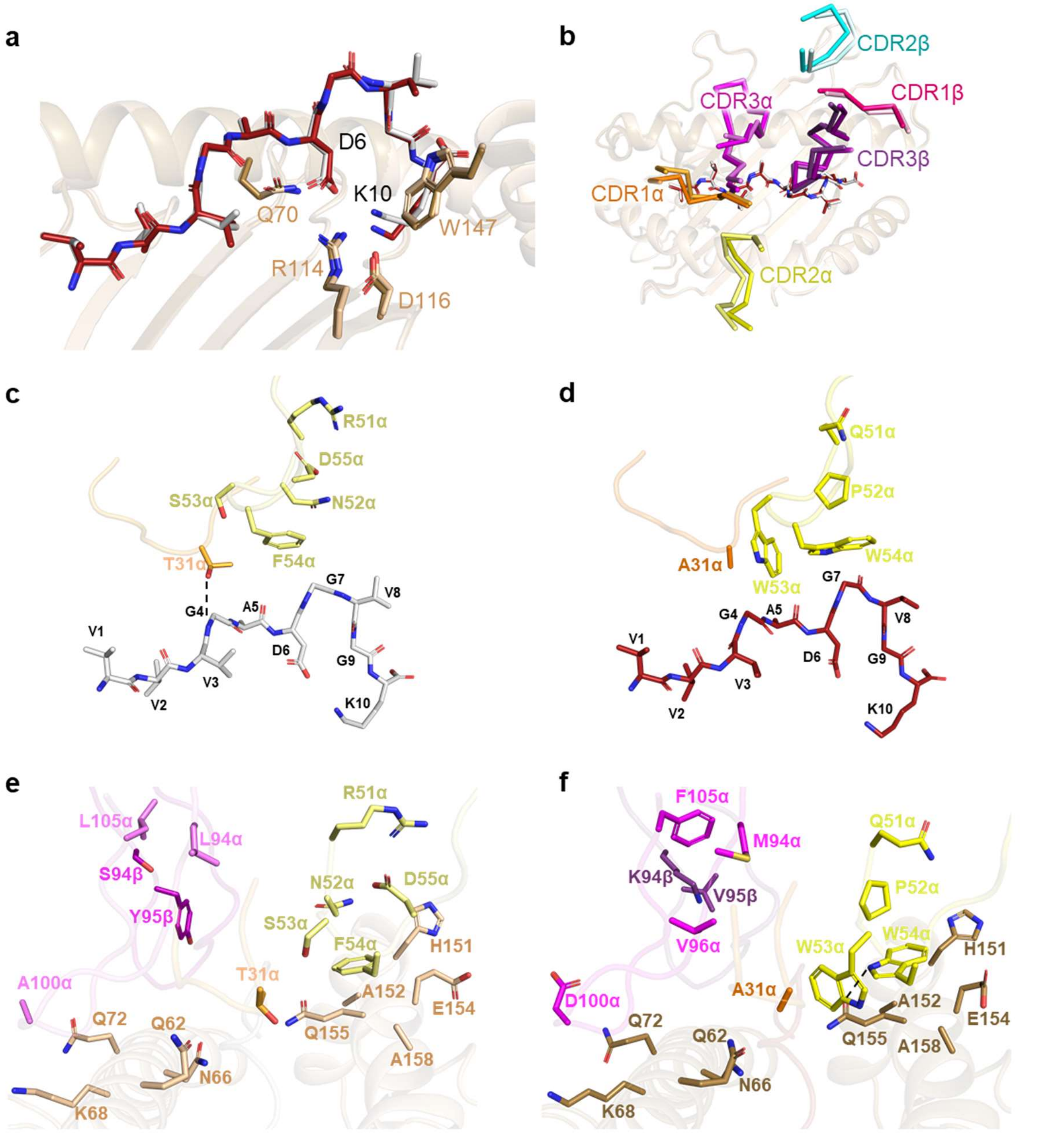

Fig S4. Comparison of JDIawtbwt- HLA-A*11-KRAS^{G12D} (PDB 7PB2) and JDIa41b41- HLA-A*11-KRAS^{G12D} (PDB 7OW6) complex structures. a. Peptide D6 and surrounding HLA residues are highlighted. Peptide sticks are shown in grey for the JDIawtbwt- HLA-A*11-KRAS^{G12D} complex. HLA residues in darker shade correspond to the JDIa41b41- HLA-A*11-KRAS^{G12D} complex. **b.** Top view with CDRs shown as ribbons. CDRs with darker shade correspond to the JDIa41b41- HLA-A*11-KRAS^{G12D} complex. **c-d.** Residues that differ between JDIawtbwt- HLA-A*11-KRAS^{G12D} **(c)** and JDIa41b1- HLA-A*11-KRAS^{G12D} **(d)** in CDR1α and CDR2α with respect to the peptide are highlighted in sticks. **e-f.**

All CDR residues that differ between JDIawtbwt- HLA-A*11-KRAS^{G12D} **(e)** and JDIa41b1-HLA-A*11-KRAS^{G12D} **(f)** are highlighted in sticks along with HLA residues that are within 5Å.

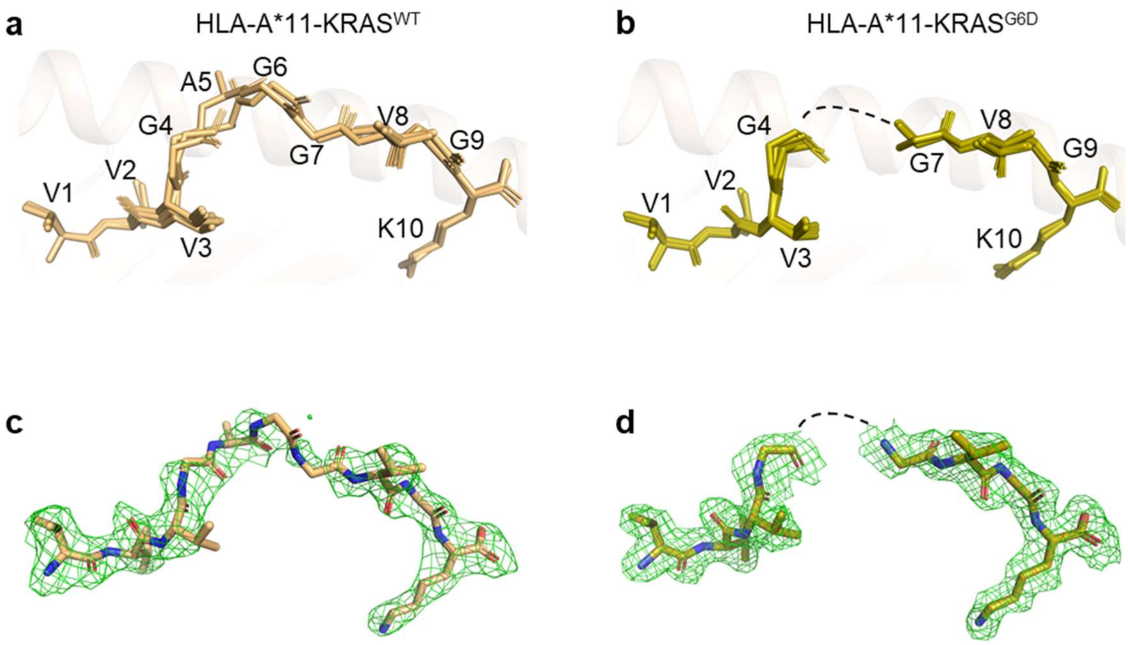

Fig S5. Comparison of peptide presentations between HLA-A*11-KRAS^{WT} (PDB 7OW3)/KRAS^{G12D} (PDB 7OW4) complexes. a. Structure of HLA-A*11-KRAS^{WT} with all four copies in the asymmetric unit superposed. The four peptide copies (in sticks) are coloured in light orange. The HLA helices are shown in cartoon **b.** Structure of HLA-A*11-KRAS^{G12D} with all four copies in the asymmetric superposed. Peptide copies (in sticks) are coloured in olive. **c.** A peptide copy (central region modelled) of the HLA-A*11-KRAS^{WT} structure with the Fo-Fc omit map at 2σ shown in green mesh. **d.** A peptide copy (missing central region indicated by dotted lines) of the HLA-A*11-KRAS^{G12D} structure with the Fo-Fc omit map at 2σ shown in green mesh.

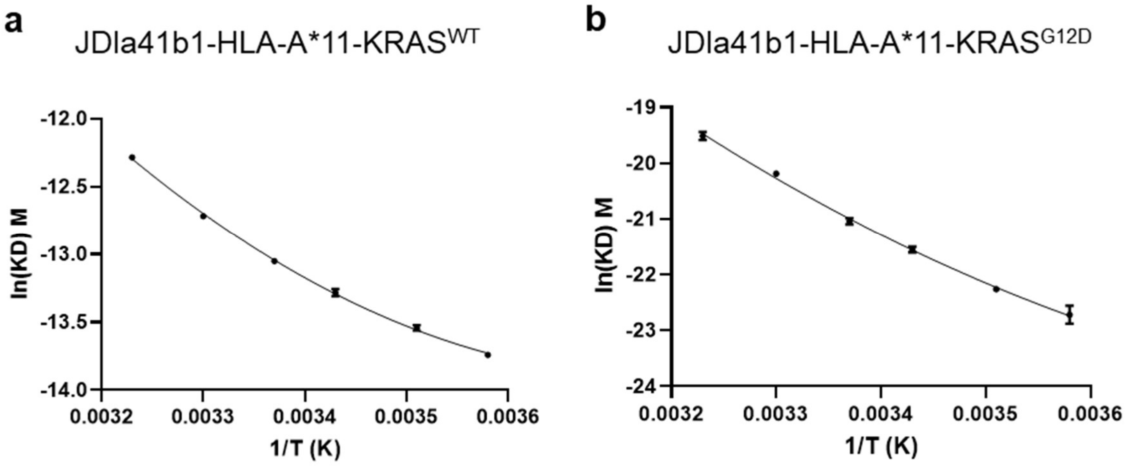

Fig S6. Thermodynamic analysis of the JDIa41b1 TCR binding to HLA-A*11-KRAS^{WT} and HLA-A*11-KRAS^{G12D}. a. JDIa41b1 TCR binding affinity (K_D) at 6°C, 12°C, 18°C, 24°C, 30°C, 36°C temperatures was used to calculate thermodynamic parameters for HLA-A*11-KRAS^{WT} and **b.** HLA-A*11-KRAS^{G12D} using non-linear Van't Hoff plots. Data (n=2) are presented as mean ± SD. Source data are provided as a Source Data file.

a

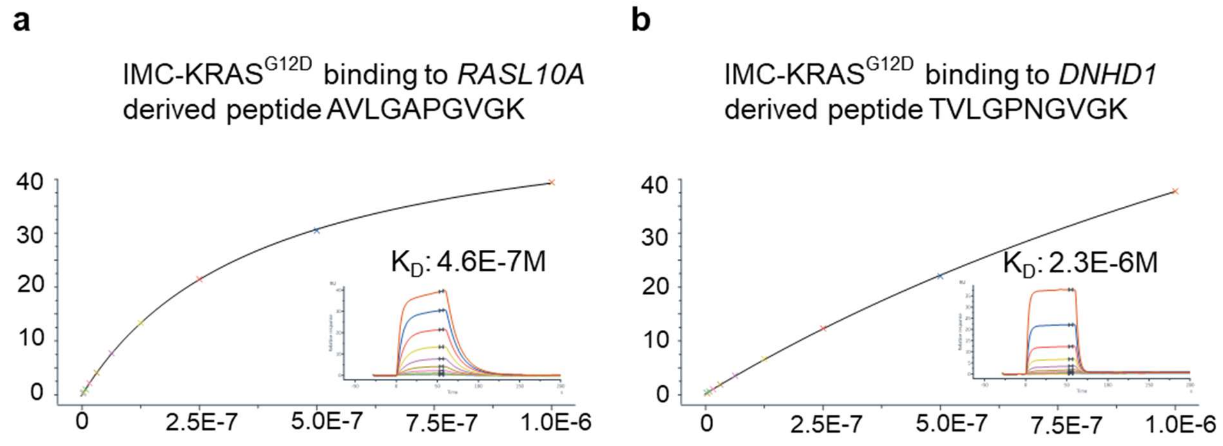

Fig S7. Affinity measurement of IMC-KRAS^{G12D} TCR binding to mimetic peptides derived from (a) *RASL10A* and (b) *DNHD1* identified based on the binding motif obtained from scHLA library screening

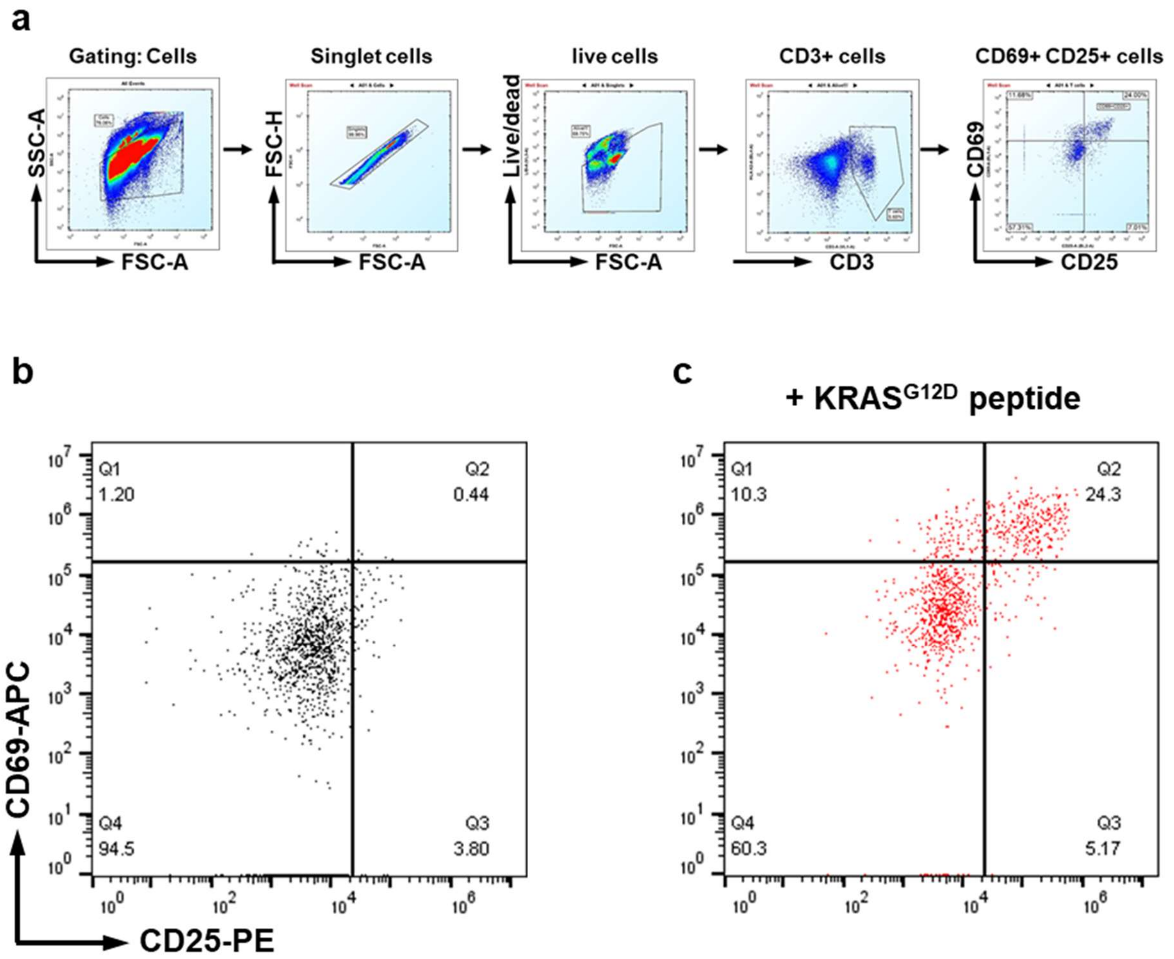

Fig S8. IMC-KRAS^{G12D} mediates T cell activation of cells presenting KRAS^{G12D} peptide in vitro. CD69 and CD25 expression was determined by flow cytometry. **(a)** Exemplification of gating strategy used. SUP-B15 cells treated with IMC-KRAS^{G12D} and co-cultured with PBMC for 24 h in the absence **(b)** or presence of 10 μM KRAS^{G12D} peptide (**c**). Data shown are from one representative experiment from two independent experiments performed (n=2). Source data are provided as a Source Data file.

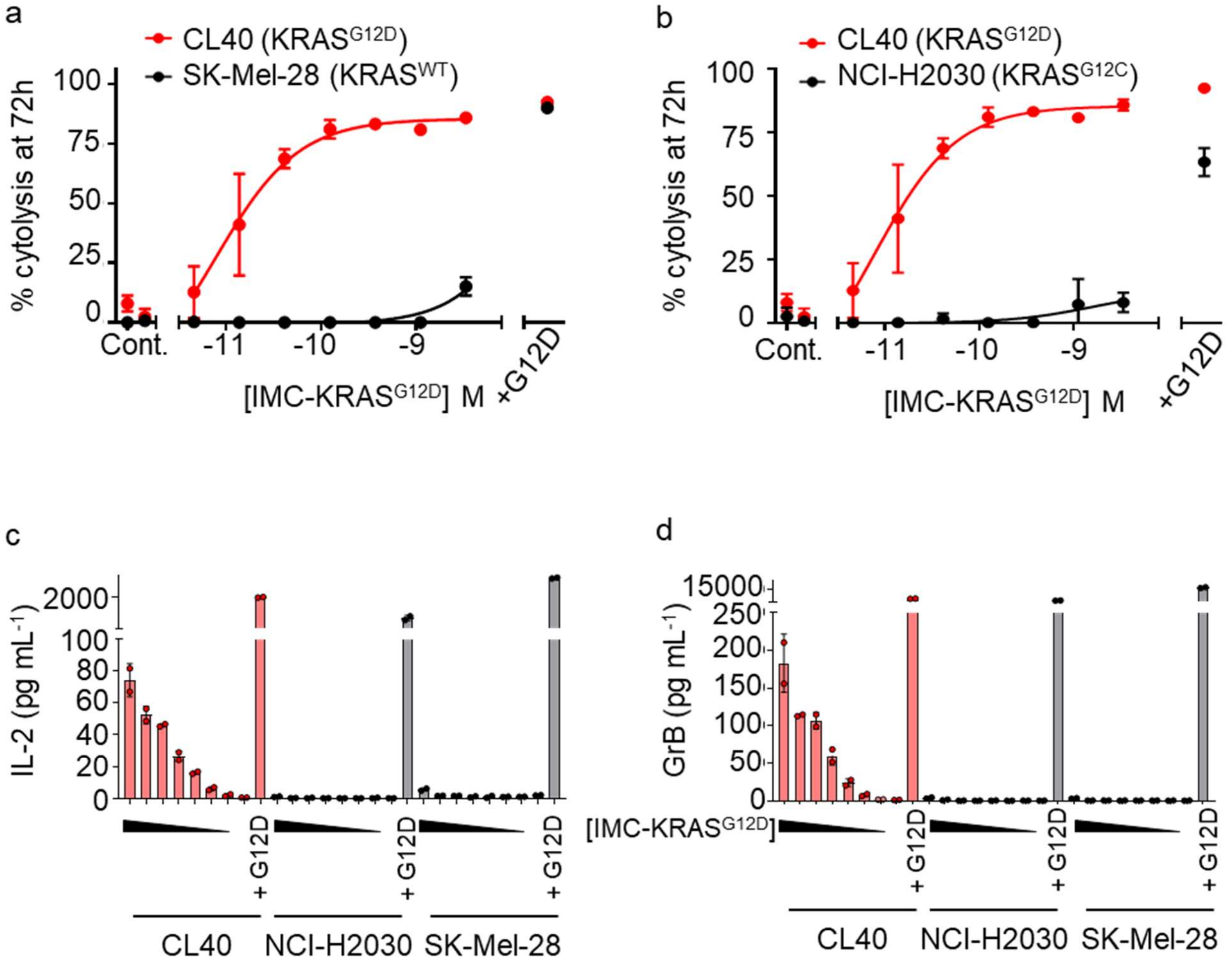

Fig S9. IMC-KRAS^{G12D} mediates redirected T cell killing, IL-2 and Granzyme B (GrB) release of KRAS^{G12D} CL40 but not KRAS^{WT} SK-Mel-28 or KRAS^{G12C} NCI-H2030 cancer cell lines in vitro. Redirected T cell killing assay of HLA-A*11+/KRAS^{G12D} CL40, HLA-A*11+/KRAS^{WT} SK-Mel-28 **(a)** or NCI-H2030 **(b)** cancer cells expressing nuclear-restricted mKATE2, treated with IMC-KRAS^{G12D} and co-cultured with PBMC for 72 h. Cells were pulsed with 10 μM KRAS^{G12D} (+G12D) peptide as a positive control. Targets alone, PBMC alone or no IMC-KRAS^{G12D} negative controls (Cont.) were performed. Assays were performed in duplicate with standard deviation shown as error bars and results confirmed using PBMC from multiple donors. Mean data of 2 biological replicates ± SD from one representative experiment from two independent experiments (n=2) is show **(c)** IL-2 and **(d)** GrB ELISA assay supernatants of killing assays shown in (Fig S9a and b) from HLA-A*11-KRAS^{G12D}+ CL40 and HLA-A*11-KRAS^{G12D}- cancer cell lines SK-

Mel-28 and NCI-H2030 treated with 3.3, 1.1, 0.37, 0.12, 0.04, 0.014, 0.005 or 0 nM IMC-KRAS^{G12D} and co-cultured with PBMC for 48 h. Cell lines were pulsed with 10 μM KRAS^{G12D} (+ G12D) peptide as a positive control. Mean data of 2 biological replicates ± SD from one representative experiment from two independent experiments (n=2) is shown. Source data are provided as a Source Data file.

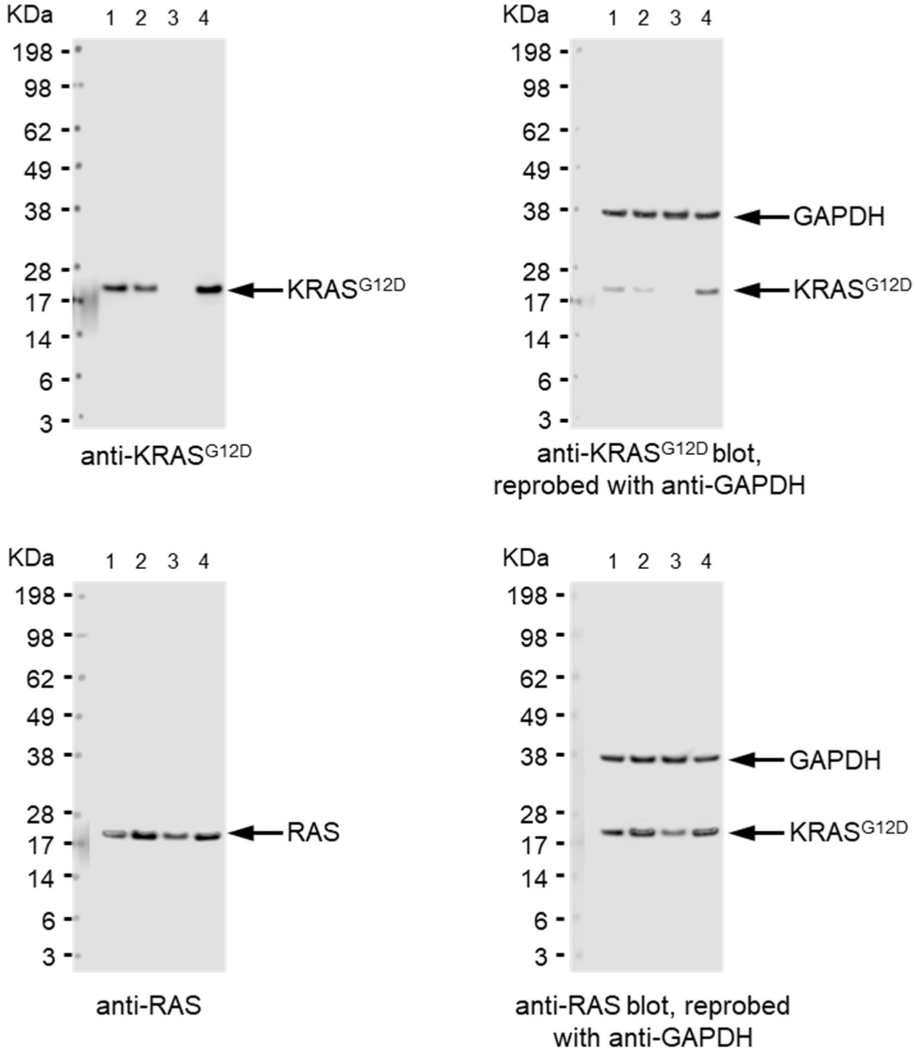

FIG S10. Uncropped scanned western blot membranes from images shown in Fig 5C (n=1). Lane order: **1**. HLA-A*11-KRAS^{G12D} clone 1, **2**. HLA-A*11-KRAS^{G12D} clone 2, **3**. HLA-A*11-KRAS^{WT} **4**. HLA-A*24-KRAS^{G12D}. Figure S10 is the source data for Fig 5C.

SUPPLEMENTAL TABLES

	Gene	Peptide
A11*01 pool1	*PSMD8*	GSYNKVFLAK
	TMCO1	SIFDGRVVAK
	RPS7	LVFPSEIVGK
	BCHE	HVYDGKFLAR
	VIM	SLPLVDTHSK
	PSMD14	AAMLDTVVFK
	MED23	ALADFLPVMK
	AAMP	ATGDMSGLLK
	DERL1	ATVAVPLVGK
	MAP4K4	ATYYGAFIKK
A11*01 pool2	*ATP5F1B*	AVDPLDSTSR
	SEC11C	AVMGAYVLLK
	SNAPIN	GIYPPGSPGK
	AKR1C2	GTYAPAEVPK
	PSMC1	ILYGPPGTGK
	KDM1B	IQFNPPLSEK
	RPS6	ISFPATGCQK
	EEF1G	KAFNQGKIFK
	PSMC5	LLYGPPGTGK
	RPS27A	QIFVKTLTGK
A11*01 pool3	*PPFIBP1*	SSFGRGFFK
	DYNLRB1	SLMHSFILK
	EXT2	SVFGTMPLK
	TOMM5	RVTPFILKK
	BEST1	ATFTGILGK
	RIOK2	STIPPELVK
	HINT1	TIFGKIIRK
	SLC25A5	SVQGIIIYR

Supplementary Table S1. Pool of unrelated peptides used to assess broad crossreactivity of parent JDI TCR. Peptides selected to prepare these pools are from ubiquitously expressed genes and predicted to be presented on HLA-A*11:01. These pools of pHLA were used to eliminate broadly cross-reactive TCRs during TCR identification stage.

Contacts	JDIa41b1-HLA-A*11-KRAS^{WT}	JDIa41b1-HLA-A*11-KRAS^{G12D}
TCR/pHLA[a]	29/3/101	31/3/106
TCRα/pHLA[a]	21/2/64	22/2/69
TCRβ/pHLA[a]	8/1/37	9/1/37
TCR/peptide[a]	6/0/22	6/0/22
TCR/HLA[a]	23/3/79	25/3/84
BSA on peptide (Å^2)[b]	223	213
BSA on HLA (Å^2)[b]	779	782
Crossing angle (°)	50.2	49.7

Supplementary Table S2. Summary of JDIa41b1-HLA-A*11-KRAS^{WT} and JDIa41b1-HLA-A*11-KRAS^{G12D} complex structures.

[a]Number of hydrogen bonds (H-bond)/salt bridges/van derWaals (vdW) (4 A°) contacts calculated with CONTACT program from the CCP4 package.

[b]Buried surface area (BSA) of TCR–peptide/TCR–HLA calculated with Molecular Operating Environment (MOE) from CCG.

Differences in HLA

Residue	Difference / Kcal. Mol^{-1}	P value
Glu58	-1.743	0.029396
Arg114	-0.5484	<0.000001
Arg75	-0.5139	0.334015
**Gln155**	-0.4308	0.000003
Lys146	-0.3133	0.000185
**Ala158**	-0.2845	<0.000001
Gln72	-0.2762	0.083838
Gln62	0.9173	0.000236
His151	0.4971	<0.000001
Asp74	0.4945	<0.000001
Glu63	0.4349	<0.000001
Ala150	0.308	<0.000001
Asp116	0.2925	<0.000001

Differences in the peptide

Residue	Difference / Kcal. Mol^{-1}	P value
**Asp6**	-0.4664	<0.000001
Gly7	-0.3422	<0.000001
Gly9	-0.326	<0.000001
**Gly4**	-0.3206	<0.000001
Val1	-0.2835	<0.000001

Differences in TCRα

Residue	Difference / Kcal. Mol^{-1}	P value
Arg50	-2.013	<0.000001
Lys70	-0.8763	0.000008
Asp29	-0.5778	0.354507
Tyr103	-0.4821	0.001073
**Trp53**	-0.3699	<0.000001
Ser95	-0.3584	0.037827

Pro97	-0.3194	0.000499
**<u>Pro52</u>**	-0.3164	<0.000001
Arg28	1.752	0.059256
	Differences in TCRβ	
Residue	Difference / Kcal. Mol^{-1}	P value
**<u>Lys94</u>**	-1.306	0.00351
His100	-1.243	<0.000001
Asn101	-0.6912	<0.000001
Gln99	-0.6125	<0.000001
Glu30	0.9333	0.01128
Pro97	0.3202	0.002327

Supplementary Table S3. Differences in contribution to the binding energy between WT and G12D bound TCR-pHLA complexes. Differences were calculated from decomposed per residue MMPBSA by subtracting the average JDIa41b1-HLA-A*11-$KRAS^{WT}$ values from the average JDIa41b1-HLA-A*11-$KRAS^{G21D}$ values. P values were determined using multiple unpaired two-tailed T-tests on GraphPad Prism 9 and values with $P<0.01$ are considered significant. TCR residues in bold and underlined are affinity-enhanced variants. HLA or peptide residues in bold and underlined are contacted by affinity-enhanced residues in the TCR. Values shown are limited to those with a difference of over ±0.25 Kcal. Mol^{-1}.

TCR-pHLA complex	HID Tautomerisation States	HIE Tautomerisation States
JDIa41b1-HLA-A*11-KRAS^{G21D}	HLA: 93, 260<br>β2M: 31<br>TCRα:<br>TCRβ: 208	HLA: 3, 151, 188, 191, 192, 197, 263<br>β2M: 13, 51, 84<br>TCRα:<br>TCRβ: 29, 47, 100, 105, 155, 168
JDIa41b1-HLA-A*11-KRAS^{WT}	HLA: 93, 260,<br>β2M: 31<br>TCRα:<br>TCRβ: 208	HLA: 3, 151, 188, 191, 192, 197, 263<br>β2M: 13, 51, 84<br>TCRα:<br>TCRβ: 29, 47, 100, 105, 155, 168

Supplementary Table S4. Histidine tautomerisation states. Assigned histidine tautomerisation states for molecular dynamic simulations of the JDIa41b1-HLA-A*11-KRAS^{WT} and JDIa41b1-HLA-A*11-KRAS^{G21D} complexes. HID corresponds to histidine residues which are singly protonated on the delta nitrogen (Nδ1) and HIE corresponds to histidine residues which are singly protonated on the epsilon nitrogen (Nε2).

	Gene expression (FPKM)					
gene	Normal colonic cells	SUP-B15	SK-Mel-28	NCI-H2030	GTEX median	GTEX High
KRAS	10.9	16.3	13.2	7.5	7.5	15.5 (blood)
DNHD1	1.0	3.8	4.0	2.3	5.0	9.7 (Spleen)

Supplementary Table S5. Gene expression in selected cells

Cell line	Supplier	Catalogue number	Method of authentication*	Date of authentication
CL40	DSMZ	ACC535	STR	07 MAY 2020
SK-Mel-28	ICLC	99010	STR	03 DEC 2019
NCI-H2030	ATCC	CRL-5914	STR	01 JUL 2021
SUP-B15	ECACC	15081302	STR	07 JAN 2022
PSN-1	ECACC	94060601	STR	31 JAN 2022
PANC-1	IZSBS	BSTCL49	STR	05 JUL 2018
Normal cell lot	Supplier	Catalogue number		
Normal Colon epithelial	ScienCell	2950	n/a**	
Pulmonary Fibroblast	Promocell	C-12360	n/a**	
Cardiac Myocyte	Promocell	C-12810	n/a**	
Cardiac Smooth	Promocell	C-12511	n/a**	
Aortic endothelial	Promocell	C-12221	n/a**	

Supplementary Table S6. Cell lines and normal cells used in this study

*Performed by ATCC
**STR validation of normal cells not applicable

Antibody	Supplier	Catalogue number	Assay (Figure)	Dilution
Rabbit anti-RAS (D2C1)	Cell Signaling Technology, MA	8955	western blot (Fig 5c and Fig S10)	1:1500
Rabbit anti-RAS^{G12D} (D8H7)		14429		1:625
Goat polyclonal HRP-conjugated anti-Rabbit		7074		1:1000
horse polyclonal HRP-conjugated anti-mouse		7076		1:5000
Mouse anti-GAPDH	Merck Millipore, MA	MAB374		1:7500
BV421 conjugated anti-CD3	Biolegend, CA	317344	flow cytometry (Fig S8)	1:100
PE conjugated anti-CD25		302606		1:100
APC conjugated anti-CD69		310910		1:100

Supplementary Table S7. Antibodies used in this study

SUPPLEMENTAL METHODS

System preparation for X-ray crystallography

The X-ray structures (JDIa41b1-HLA-A*11-KRASWT and JDIa41b1-HLA-A*11-KRAS^{G12D}) were used as starting points for MD simulations. An extra water molecule was introduced into the WT structure at the position of the mutated aspartate oxygen atom. Missing residues in the JDIa41b1-HLA-A*11-KRAS^{G12D} structure were copied from the equivalent JDIa41b1-HLA-A*11-KRAS^{WT} structure after aligning the co-ordinates of surrounding regions using Modeller (1). MolProbidity (2) was used to assign asparagine and glutamine rotamers as well as histidine tautomers (Table S3). PropKa 3.0 (3) was used to determine protonation states, resulting in the protonation of D77 in chain A and H138 in chain E. Structures, with crystal waters retained, were solvated in a water box with minimum of 10 Å between any protein atoms and the edge of the box. Simulations were performed using Amber18 (4) with proteins described by the ff14SB forcefield (5) and water molecules described by the TIP3P water model. Sodium ions were added to neutralise the solvent.

Structure equilibration procedure

Prior to production simulations, a minimising, heating, and equilibration procedure was performed as follows. In all simulations, a temperature of 298 K and pressure of 1 atm were used in the NPT ensemble. All dynamics steps had the SHAKE algorithm applied to constrain hydrogen containing bonds. An 8 Å direct space non-bonded cut off was used throughout with long range electrostatics evaluated using the particle mesh Ewald algorithm (6). Temperature was controlled with langevin temperature control (1 ps-1 collision frequency) and pressure was controlled using the Berendsen barostat (1 ps relaxation time). An initial solvent minimisation was performed using 500 steps of steepest descent followed by 500 steps of conjugate gradient. Restraints were applied to all heavy atoms (10 Kcal. Mol-1. Å-1). The solvent was then rapidly heated from 50 K to 298 K, with the heavy atom restraints maintained. This was done over 200 ps using the NVT ensemble with a 1 fs timestep. A second minimisation was performed, using 5 Kcal. Mol-1. Å-1 restraints on only the Cα carbon atoms. 500 steps of steepest descent followed by 500 steps of conjugate gradient were again used. Keeping the Cα carbon atom restraints, another heating step was performed from 25 K to 298 K over 50 ps in the NVT ensemble, with a 1 fs timestep. The restraints were gradually removed over a series of 4 10 ps simulations, reducing by 1 Kcal. Mol-1. Å-1 each simulation, followed by a final 1 ns simulation with no restraints. These

were all performed in the NPT ensemble with a 2 fs timestep. Finally, 4 ns production simulations were performed, saving frames every 10 ps, in the NPT ensemble with a 2 fs timestep. 25 independent repeats were performed.

Electrostatics

The aPBS PyMOL plugin (8) was used to determine the change in electrostatic potential in the WT and mutant bound HLA structures. A grid spacing value of 0.25 was used with the rest of the settings left at their default values.

Flow cytometry

BV421 conjugated anti-CD3 (Biolegend, CA, catalogue #317344), APC congujated anti-CD69 (Biolegend #310910) and PE conjugated anti-CD25 (Biolegend #302606) antibodies were used for flow cytometric analysis. To examine cell viability and exclude dead cells, LIVE/DEAD^{TM} Yellow (Invitrogen, L34959) was used. For staining, antibodies were diluted in PBS FACS buffer containing 2% foetal bovine serum and 2 mM EDTA supplemented with human TruStain FcX (Biolegend #422302) and incubated in the dark at 4oC for 30 mins. Stained samples were washed three times with FACS buffer prior to data acquisition. Data were acquired on a Intellicyt flow cytometer (Sartorius, Germany), compensated using anti-mouse Ig k, negative control compensation particles (BD #552843) and ArC Amine Reactive compensation bead kit (Invitrogen A10346) and analysed using FlowJo software (version 10.7.1 ,FlowJo LLC / BD).

Electrochemiluminescent ELISA

Cell culture supernatants were analysed for the levels of IL-2 and Granzyme B using multiplex Meso Scale Discovery assays and a QuickPlex Imager plate reader and Discovery wokbench software in accordance with the manufacturer’s instructions (MSD, Rockville, MD).

SUPPLEMENTAL REFERENCES

1. Webb B, Sali A. Comparative Protein Structure Modeling Using MODELLER [Internet]. *Curr. Protoc. Bioinforma.* 2014;47(1):5.6.1-5.6.32.

2. Chen VB et al. *MolProbity* : all-atom structure validation for macromolecular crystallography [Internet]. *Acta Crystallogr. Sect. D Biol. Crystallogr.* 2010;66(1):12–21.

3. Søndergaard CR, Olsson MHM, Rostkowski M, Jensen JH. Improved Treatment of Ligands and Coupling Effects in Empirical Calculation and Rationalization of pKa Values. [Internet]. *J. Chem. Theory Comput.* 2011;7(7):2284–95.

4. Song LF, Lee T-S, Zhu C, York DM, Merz KM. Using AMBER18 for Relative Free Energy Calculations [Internet]. *J. Chem. Inf. Model.* 2019;59(7):3128–3135.

5. Maier JA et al. ff14SB: Improving the Accuracy of Protein Side Chain and Backbone Parameters from ff99SB. [Internet]. *J. Chem. Theory Comput.* 2015;11(8):3696–713.

6. Darden T, York D, Pedersen L. Particle mesh Ewald: An $N\cdot\log(N)$ method for Ewald sums in large systems [Internet]. *J. Chem. Phys.* 1993;98(12):10089–10092.

7. Roe DR, Cheatham TE. PTRAJ and CPPTRAJ: Software for Processing and Analysis of Molecular Dynamics Trajectory Data [Internet]. *J. Chem. Theory Comput.* 2013;9(7):3084–3095.
