# Supplementary material for: Therapeutic high affinity T cell receptor targeting a KRASG12D cancer neoantigen
Source: Nat Commun. 2022 Sep 10;13:5333. doi: 10.1038/s41467-022-32811-1 (PMC9464187; doi:10.1038/s41467-022-32811-1)
Supplement: Supplementary file 3 — Reporting Summary [file 41467_2022_32811_MOESM3_ESM.pdf]

nature portfolio

Corresponding author(s): NCOMMS-21-35973C

Last updated by author(s): Aug 9, 2022

Reporting Summary

Nature Portfolio wishes to improve the reproducibility of the work that we publish. This form provides structure for consistency and transparency in reporting. For further information on Nature Portfolio policies, see our Editorial Policies and the Editorial Policy Checklist.

Statistics

For all statistical analyses, confirm that the following items are present in the figure legend, table legend, main text, or Methods section.

n/a	Confirmed	
☐	☒	The exact sample size (*n*) for each experimental group/condition, given as a discrete number and unit of measurement
☒	☐	A statement on whether measurements were taken from distinct samples or whether the same sample was measured repeatedly
☐	☒	The statistical test(s) used AND whether they are one- or two-sided<br>*Only common tests should be described solely by name; describe more complex techniques in the Methods section.*
☒	☐	A description of all covariates tested
☒	☐	A description of any assumptions or corrections, such as tests of normality and adjustment for multiple comparisons
☐	☒	A full description of the statistical parameters including central tendency (e.g. means) or other basic estimates (e.g. regression coefficient) AND variation (e.g. standard deviation) or associated estimates of uncertainty (e.g. confidence intervals)
☒	☐	For null hypothesis testing, the test statistic (e.g. *F*, *t*, *r*) with confidence intervals, effect sizes, degrees of freedom and *P* value noted<br>*Give P values as exact values whenever suitable.*
☒	☐	For Bayesian analysis, information on the choice of priors and Markov chain Monte Carlo settings
☒	☐	For hierarchical and complex designs, identification of the appropriate level for tests and full reporting of outcomes
☒	☐	Estimates of effect sizes (e.g. Cohen's *d*, Pearson's *r*), indicating how they were calculated

Our web collection on statistics for biologists contains articles on many of the points above.

Software and code

Policy information about availability of computer code

Data collection	ELISPOT assays were imaged and quantified using the the CTL ImmunoSpot Analyzer and ImmunoSpot® software; Western blots were detected using a Li-Cor C-digit scanner and Image Studio software,
Data analysis	Dose response curves were analysed using GraphPad Prism V. 8; version 8 or 9 was used in the preparation of Figures and version 9 was used to analyse MD data; Thermodynamics data was analysed using the Thermodynamics wizard in Biacore T200 Evaluation Software 3.1; X-Ray diffraction images were indexed, integrated, scaled, and merged using the autoproc pipeline implementing XDS/XSCALE and POINTLESS/AIMLESS or using xia2 automated pipeline implementing DIALS. Crystal structures were analysed using Phaser, COOT, Refmac / CCP4 suite, PDB-REDO, Molprobity and PyMOL; Molecular dynamics simulations were performed with MMPBSA.py.MPI script used on Amber16

For manuscripts utilizing custom algorithms or software that are central to the research but not yet described in published literature, software must be made available to editors and reviewers. We strongly encourage code deposition in a community repository (e.g. GitHub). See the Nature Portfolio guidelines for submitting code & software for further information.

Data

Policy information about availability of data

All manuscripts must include a data availability statement. This statement should provide the following information, where applicable:

- Accession codes, unique identifiers, or web links for publicly available datasets
- A description of any restrictions on data availability
- For clinical datasets or third party data, please ensure that the statement adheres to our policy

The atomic coordinates and experimental data (codes 7OW3, 7OW4, 7PB2, 7OW5, 7OW6) have been deposited in the Protein Data Bank (www.wwpdb.org).

Field-specific reporting

Please select the one below that is the best fit for your research. If you are not sure, read the appropriate sections before making your selection.

☒ Life sciences ☐ Behavioural & social sciences ☐ Ecological, evolutionary & environmental sciences

For a reference copy of the document with all sections, see nature.com/documents/nr-reporting-summary-flat.pdf

Life sciences study design

All studies must disclose on these points even when the disclosure is negative.

Sample size	n/a - no sample size calculations were perfoformed
Data exclusions	n/a - no data were excluded
Replication	Biacore and cell data presented in Figures 1a,c,d (unless indicated) and 5c are n=1 and the findings are supported by subsequent experiments; Biacore and thermodynamic analysis represent n=2 independent experiments; Cell-based assay data shown in Figures 1b, 4a,e and 5 a,b,d,e,f are representative experiments performed on n=2 independent occasions. Results were confirmed using PBMC from multiple donors. The data shown are duplicate or triplicate technical replicates with standard deviation or stand error of mean shown as error bars
Randomization	n/a
Blinding	n/a

Reporting for specific materials, systems and methods

We require information from authors about some types of materials, experimental systems and methods used in many studies. Here, indicate whether each material, system or method listed is relevant to your study. If you are not sure if a list item applies to your research, read the appropriate section before selecting a response.

Materials & experimental systems

n/a	Involved in the study	
☐	☒	Antibodies
☐	☒	Eukaryotic cell lines
☒	☐	Palaeontology and archaeology
☒	☐	Animals and other organisms
☒	☐	Human research participants
☒	☐	Clinical data
☒	☐	Dual use research of concern

Methods

n/a	Involved in the study	
☒	☐	ChIP-seq
☐	☒	Flow cytometry
☒	☐	MRI-based neuroimaging

Antibodies

Antibodies used	Rabbit anti-RAS (#8955) , RAS G12D (#14429) and HRP-conjugated secondary antibodies (#7074 & 7076) were purchased from Cell Signaling Technology (Danvers, MA). Anti-GAPDH (MAB374) was purchased from Merck Millipore (MA). BV421 conjugated anti-CD3 (Biolegend, CA, catalogue #317344), APC congujated anti-CD69 (Biolegend #310910) and PE conjugated anti-CD25 (Biolegend #302606) antibodies were used for flow cytometric analysis. Antibody dilutions are detailed in the supplementary information.
Validation	Antibodies were validated for western blot or flow cytometry by the suppliers

Eukaryotic cell lines

Policy information about cell lines

Cell line source(s)	Cell lines and normal cell lots were obtained from commercial suppliers: CL40 (DSMZ), NCI-H2030 (ATCC), SUP-B15 and PSN-1 (ECACC), PANC-1 (IZSBS), SK-Mel-28 (ICLC), Normal colon epithelial cells (Sciencell), pulmonary fibroblasts, cardiac myocytes, cardiac smooth muscle and aortic endothelial cells (Promocell)
Authentication	cell lines were validated with STR analysis performed by ATCC
Mycoplasma contamination	all cell lines tested negative for the presence of mycoplasma; normal human colonic epithelial cells were tested by the supplier for the presence of mycoplasma and the results were negative.

Commonly misidentified lines
(See ICLAC register)

n/a - none of the cell lines used in this study are on the ICLAC register of commonly misidentified lines

Flow Cytometry

Plots

Confirm that:

☒ The axis labels state the marker and fluorochrome used (e.g. CD4-FITC).

☒ The axis scales are clearly visible. Include numbers along axes only for bottom left plot of group (a 'group' is an analysis of identical markers).

☒ All plots are contour plots with outliers or pseudocolor plots.

☒ A numerical value for number of cells or percentage (with statistics) is provided.

Methodology

Sample preparation	Commercially sourced cryofrozen PBMC and well characterized cell lines from reputable commercial suppliers were used.
Instrument	Intellicyt flow cytometer (Sartorius, Germany)
Software	FlowJo software (version 10.7.1 ,FlowJo LLC / BD)
Cell population abundance	n/a
Gating strategy	FSC/SSC was used to define the initial cell population. A singlet cell gate from then set from FSC-H/FSC-A followed by a live-cell gate set using LIVE/DEAD Yellow (Invitrogen, L34959). The CD3+ population of live cells was then analysed for expression of CD69 and CD25 as shown in Supp Fig 8

☒ Tick this box to confirm that a figure exemplifying the gating strategy is provided in the Supplementary Information.
